# Exploring Policies, Strategies, and Legislations Related to the One Health Approach to Zoonoses, Antimicrobial Stewardship, and Climate Change in Jordan: A Multimethod Study with SWOT Analysis

**DOI:** 10.3390/ijerph22050749

**Published:** 2025-05-09

**Authors:** Dalia K. Zayed, Salam Momani, Muna Horabi, Alaa Alquran, Furat K. Al-Nawaiseh, Ala Bin Tarif, Omar F. Nimri, Mohammad S. Alyahya, Thaira Madi, Ahmad Shatat, Mayes Alahmad, Tuqa Jomhawi, Bassam Hijjawi, Adel Belbiesi, Ala’a B. Al-Tammemi

**Affiliations:** 1 Research, Policy, and Training Directorate, Jordan Center for Disease Control, Amman 11183, Jordan; d.zayed@jcdc.gov.jo (D.K.Z.); m.horabi@jcdc.gov.jo (M.H.); 2 Disease Prevention and Control Directorate, Jordan Center for Disease Control, Amman 11183, Jordan; s.momani@jcdc.gov.jo (S.M.); a.tareif@jcdc.gov.jo (A.B.T.); o.nimri@jcdc.gov.jo (O.F.N.); 3 Epidemiological Surveillance Directorate, Jordan Center for Disease Control, Amman 11183, Jordan; a.mahmoud@jcdc.gov.jo (A.A.); f.nawaiseh@jcdc.gov.jo (F.K.A.-N.); 4 Jordan Center for Disease Control, Amman 11183, Jordan; msalyahya@just.edu.jo (M.S.A.); b.hijawi@jcdc.gov.jo (B.H.); a.belbaisi@jcdc.gov.jo (A.B.); 5 Department of Health Management and Policy, Faculty of Medicine, Jordan University of Science and Technology, Irbid 22110, Jordan; 6 Accreditation Department, Health Care Accreditation Council, Amman 11181, Jordan; talmadi@hcac.com.jo (T.M.); tjamhawi@hcac.com.jo (T.J.); 7 Survey and Surveyors Unit, Health Care Accreditation Council, Amman 11181, Jordan; ashatat@hcac.com.jo (A.S.); malahmad@hcac.com.jo (M.A.)

**Keywords:** Jordan, mapping, climate change, AMR, AMS, One Health, zoonoses, policies, strategies

## Abstract

**Background:** Mapping policies, strategies, and legislations related to disease prevention in Jordan is pivotal for strengthening the country’s public health infrastructure. The aims of our study were to identify, review, and map the existing national policies, strategies, and legislations related to the One Health approach to zoonoses, antimicrobial stewardship (AMS), and climate change in Jordan. Additionally, we identified the key strengths and major gaps and uncovered opportunities for enhancement. The current paper reports a part of a nationwide project which was jointly executed in 2023 by the Jordan Center for Disease Control and the Health Care Accreditation Council. **Methods:** A multimethod approach was employed, including a comprehensive desk review of any existing policies, strategies, and legislations, along with key informant interviews involving key stakeholders. The combination of the desk review and key informant interviews allowed for a more nuanced understanding of the gaps, strengths, and challenges in Jordan’s approach to One Health, AMS, and climate change adaptation. By triangulating the findings from both methods, the study was able to cross-validate its results and ensure greater reliability and accuracy in its conclusions. **Results:** Our analyses revealed that Jordan has made notable progress in integrating the One Health approach within its regulatory framework, particularly in managing zoonotic diseases, AMS, and climate change. Nevertheless, there is a need for more explicit and effective intersectoral coordination. While the country’s AMS initiatives are supported by a national action plan, they are limited by inadequate public awareness, veterinary regulations, and monitoring systems. Moreover, Jordan’s climate change strategies, aligned with broader sustainability goals and integrated into national frameworks like the environmental protection law, are constrained by a lack of emergency preparedness and multisectoral collaboration. The SWOT analysis highlighted strengths, including robust legal structures and international collaborations, while identifying gaps in enforcement and the need for updated guidelines. Opportunities exist to enhance the reporting mechanisms, public awareness, and international partnerships. **Conclusions:** Jordan’s integration of the One Health approach to zoonotic diseases, AMS, and climate change adaptation into its disease prevention policies is commendable and aligns with global health priorities. To further enhance these initiatives, Jordan could benefit from updating its public health law and the relevant guidelines and policies, strengthening and structuring public awareness campaigns, and developing detailed climate change adaptation strategies.

## 1. Introduction

The One Health approach is a transdisciplinary multisectoral strategy that aims to achieve optimal health outcomes while recognizing the interconnectedness of people, animals, plants, and their common environments [1,2,3]. The World Health Organization (WHO) established the One Health initiative to unify the work on human, animal, and environmental health throughout various stakeholders, and in collaboration with the Food and Agriculture Organization of the United Nations (FAO), the United Nations Environment Program (UNEP), and the World Organization for Animal Health (WOAH) as part of the One Health Quadripartite [4]. The quadripartite promotes cross-sectoral methods to reduce health risks at the human–animal–ecosystem interface. Developing a One Health strategy is crucial to moving forward in combatting antimicrobial resistance, ensuring food safety, preventing environmental risks to human and animal health, and anticipating, preventing, detecting, and controlling diseases that move between humans and animals (zoonotic diseases) [5]. In order to manage disease outbreaks and lessen the burden of zoonotic diseases, the One Health approach places a strong emphasis on the value of preventative measures and early diagnoses [6], and the One Health strategy is required for the efficient control and prevention of Emerging Infectious Diseases (EIDs) [7].

The One Health approach and Antimicrobial Resistance (AMR) are strongly interconnected, as both address the complex interactions between humans, animals, and the environment. AMR arises when pathogens evolve to resist antimicrobial agents due to overuse or misuse in humans, animals, and agriculture, and they can spread through shared environments. The One Health approach promotes a collaborative, integrated strategy to monitor, prevent, and control AMR by considering these cross-sectoral links, ensuring that efforts to manage resistance encompass human health, veterinary practices, and environmental protection, thereby fostering a more comprehensive and effective response to this global health threat [8].

AMR is a global health challenge that has gained prominence in recent decades due to its complex and multifaceted nature. The rise in AMR is driven by various factors, including the imprudent and excessive use of antimicrobial agents in healthcare practices, agriculture, livestock, and the environment [9]. Other contributing factors include inadequate infection prevention and control practices, limited access to clean water and sanitation facilities, and insufficient investments in the research and development of new antimicrobial agents [10]. The WHO has recognized the seriousness of the situation and, to address the escalating AMR crisis, global initiatives in antimicrobial stewardship (AMS) have emerged [11]. AMS involves an integrated and multifaceted strategy aimed at promoting the appropriate and responsible use of antimicrobial agents. The ultimate goal of this approach is to improve patient outcomes, reduce the emergence and spread of resistant pathogens, and preserve the effectiveness of these vital therapeutic compounds [12].

Typically, AMS programs encompass a range of interventions aimed at addressing AMR. These interventions include implementing evidence-based guidelines and protocols for antimicrobial prescription, conducting regular assessments of antimicrobial utilization to provide tailored recommendations for healthcare providers, educating healthcare professionals, policymakers, and the general public about the importance of prudent antimicrobial use, strengthening infection prevention and control measures to limit the dissemination of resistant microorganisms, enhancing national and global surveillance systems to monitor AMR trends and patterns, and promoting collaboration and coordination among various stakeholders, such as healthcare institutions, regulatory agencies, and research establishments [13,14].

Additionally, the environment plays a crucial role in the emergence and evolution of new diseases and pathogens by serving as a reservoir and conduit for the transmission of microorganisms, facilitating their adaptation and spread through factors such as climate change, habitat disruption, and environmental contamination [15]. In the 21st century, there is a substantial challenge, which is the detrimental health impacts of climate change on humankind. There is an urgent need for further research to elucidate the intricate web of interactions between climate change and diverse health risks, both directly and indirectly, as a result of natural, biological, and anthropogenic factors [16]. From a One Health perspective, climate change significantly alters factors that influence disease transmission. Climate change is a loosely defined term. However, most of the literature agrees that climate change is a significant and long-lasting change in the Earth’s climate and weather patterns [17].

These changes encompass the spatial distribution of biological vectors, human encroachment, and deforestation, disruptions to animal migration patterns, evolving communal behaviors, and management practices concerning animals, crops, and their environments. Addressing climate change effectively necessitates a multifaceted approach that transcends the realm of research solely. Fostering collaboration across diverse sectors is paramount. Establishing national and international networks and platforms are crucial to strengthen stakeholder cooperation, an essential step for advancing progress in climate change mitigation and integrated health governance [16]. The overall condition of global health is significantly affected by the seasonal fluctuations observed over the year. Temperature variations, rainfall, and sunlight have direct impacts on human health.

Examining the policies, legislations, and strategies related to One Health, AMS, and climate change is crucial for understanding their interconnected impact on public health, acknowledging that these domains are interlinked and collectively influence health outcomes. The One Health framework has been widely discussed in the context of zoonotic disease control and global health governance, highlighting its effectiveness in facilitating coordinated responses to emerging threats [18]. A recent study estimated that in 2021, 1.14 million deaths were directly attributed to bacterial AMR globally, and this burden is forecasted to reach 1.91 million deaths by 2050 [19]. Effective AMS programs, as promoted by the WHO, include interventions such as evidence-based prescription guidelines, educational initiatives for healthcare providers, and robust infection control measures [20]. Additionally, climate change is acknowledged as a major driver of health risks, influencing disease transmission dynamics through shifts in vector distributions, altered habitats, and extreme weather events [21,22,23,24]. Comprehensive public health strategies that integrate these factors are essential for building resilient health systems capable of responding to multidimensional health challenges. By systematically mapping these documents, we can identify key intersections and gaps in our understanding, facilitating more effective strategies to address emerging health threats, improve disease prevention, and ultimately enhance public health resilience. Consequently, the aims of our current study were to identify, review, and map the existing national policies, strategies, and legislations related to the One Health approach to zoonoses, AMS, and climate change in Jordan, a middle-income country located in the Eastern Mediterranean Region (EMR) with a population count of 11.6 million [25]. Additionally, we identified the key strengths and major gaps and uncovered potential opportunities for enhancement and development in the future.

## 2. Materials and Methods

### 2.1. Study Design

The current paper reports a part of a broader nationwide project which was jointly executed in the period June–November 2023 by the Jordan Center for Disease Control (JCDC) and the Health Care Accreditation Council (HCAC) in Jordan. This national project is aimed at mapping and reviewing the existing national policies, strategies, and legislations related to disease prevention and control in Jordan and, additionally, to identify the strengths, challenges, and opportunities. To effectively address the complex and interconnected issues of One Health, AMS, and climate change, a multimethod approach was employed due to its ability to integrate diverse data sources and perspectives to provide a more comprehensive understanding.

**Figure 1:**
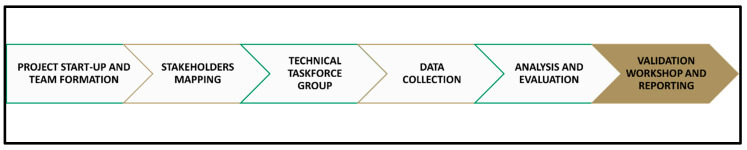
Project flowchart.

The multimethod approach in our study included a comprehensive desk review for any existing policies, strategies, and legislations related to the One Health approach to zoonoses, AMS, and climate change in Jordan and key informant interviews (KIIs) involving discussions with relevant key stakeholders and subject matter experts (SMEs) in the country. To facilitate the data collection process, an official circular indicating data sharing and the facilitation of appointments with focal points was disseminated amongst the stakeholders. The combination of the desk review and key informant interviews allowed for a more nuanced understanding of the gaps, strengths, and challenges in Jordan’s approach to One Health, AMS, and climate change adaptation. By triangulating the findings from both methods, the study was able to cross-validate its results and ensure greater reliability and accuracy in its conclusions. See Figure 1, which describes the project phases.

### 2.2. Data Collection

#### 2.2.1. Desk Review

This phase was conducted in the period of June to August 2023. In this phase, data related to the chosen subjects mentioned earlier were gathered by conducting comprehensive desk research for a wide range of documents, including policies, strategies, and legislations. The desk review identified relevant documents through a holistic search of national databases, governmental and non-governmental websites, and United Nations (UN) organizations. The focus was on national policies, strategies, and legislations directly related to disease prevention and control. Key information from each document, such as the title, publication date, issuing authorities, objectives, and policy measures, was extracted and organized into a structured database.

#### 2.2.2. Key Informant Interviews (KIIs)

During this phase, which was conducted in July and August 2023, individuals with relevant experience and expertise in the concerned fields from key stakeholders were interviewed, aiming at gaining a comprehensive understanding and insights, identifying any challenges, and providing recommendations. To ensure a comprehensive and multisectoral perspective, 41 participants from key stakeholders were involved in the KIIs. The participants were selected based on their expertise and roles within a diverse range of governmental, non-governmental, and international sectors. These included senior officials and SMEs from the Ministry of Health (e.g., Directorates of Communicable Diseases, Epidemiology, Environmental Health, the Vaccination and Serum Department, and others), Royal Medical Services, the Ministry of Agriculture (e.g., Animal Wealth Directorate), the Ministry of Environment, the Jordan Food and Drug Administration (JFDA), and the Ministry of Water and Irrigation. Representatives from the Amman Municipality and the Ministry of Labor were also included for their roles in health and occupational safety.

This study further benefited from the participation of officials from the National Center for Security and Crisis Management (NCSCM), who provided insights into public health emergency planning. To enrich the international perspective, key informants from organizations such as the World Health Organization (WHO), United Nations Children’s Fund (UNICEF), United Nations High Commissioner for Refugees (UNHCR), Food and Agriculture Organization (FAO), International Organization for Migration (IOM), and United Nations Relief and Works Agency (UNRWA) were also engaged. Additionally, input was gathered from non-governmental organizations (NGOs) involved in public health advocacy, the King Hussein Cancer Center (KHCC), and public health consultants from academic institutions such as Jordan University of Science and Technology (JUST).

These key informants held positions such as directors, department heads (e.g., Infection Control, One Health), and technical experts, ensuring that the study captured insights from various levels of policy implementation. Their contributions were critical for identifying the existing legislations, strategies, and policy documents, informing a thematic analysis that assessed the strengths, gaps, and opportunities within Jordan’s disease prevention landscape. The list of key stakeholders involved in the KIIs is provided in Supplementary File S1. The interviews were conducted in multiple stages using various methods, including Focused Group Discussions (FGDs), face-to-face interviews, and virtual interactions. A semi-structured interview guide was developed by the project’s committee (Supplementary File S2).

The main objectives of the KIIs were to collect valuable perspectives from diverse stakeholders, including experts in the field, to comprehensively evaluate the strengths and limitations of the existing policies, legislations, and strategies to identify the gaps, challenges, and limitations in the current efforts to review the mechanisms in place and collaborative efforts across sectors, and to explore expert insights to inform policy recommendations, facilitate evidence-based decision making, and contribute to improved public health outcomes in Jordan. The interviews were recorded with the consent of the participants and were utilized in the analysis. The insights from the KIIs were integrated with the data we obtained from the literature research, which helped in enriching the understanding of Jordan’s health system approach to the examined topics.

### 2.3. Situational Analysis and Benchmarking

Benchmarking is a systematic process that enables researchers to assess Jordan’s performance, policies, and practices in comparison to esteemed international organizations such as the WHO and the U.S. Centers for Disease Control and Prevention (CDC). By comparing different strengths and weaknesses to external benchmarks, established norms, and optimal performances, benchmarking offers a contextual foundation for evaluation. We were able to pinpoint internal strengths as well as shortcomings by integrating benchmarking into our study. By using a comparison approach, it was possible to identify areas for improvement and find ways to align them with the best international practices. Moreover, by examining successful strategies and best practices based on international standards, valuable insights were obtained into how Jordan can capitalize on external advantages.

Our benchmarking involved comparing the key components of Jordan’s policies with the global best practices. Key documents included the WHO Guidelines on the People-Centered Approach to Addressing Antimicrobial Resistance in Human Health [26], the One Health Tripartite Guide to Addressing Zoonotic Diseases [27], and the compendium of the WHO and other UN guidance on the Health and Environment Climate Change chapter [28]. This was followed by conducting a comparative analysis in which the researchers systematically compared the collected data from Jordan with those of the selected benchmarks. The comparative analysis highlighted areas of alignment, disparities, and potential challenges in Jordan’s policies and practices. By conducting a thorough side-by-side examination, we aimed to gain a comprehensive understanding of the strengths, challenges, and opportunities within Jordan’s approach to the examined subjects.

SWOT analysis was used in this study due to its proven effectiveness in condensing intricate policy data into a clear and manageable framework. The process of conducting the SWOT analysis started with identifying and reviewing the existing policies, strategies, and legislations related to the One Health approach to zoonoses, AMS, and climate change. The required data were collected from various sources, with support from key stakeholders and SMEs. Next, the strengths of the current system were explored, focusing on what is working well, such as effective policies, resources, and institutional support. Weaknesses were then analyzed, highlighting internal challenges like gaps in coordination or communication, resource limitations, and enforcement issues. External opportunities were explored, looking for emerging trends or collaborations that could enhance the effectiveness of the policies, while potential threats, such as political resistance or a lack of unified multisectoral data, were identified. The findings were synthesized to highlight the relationships between the strengths, weaknesses, opportunities, and threats, and recommendations were provided. The interviews with the SMEs provided a foundational framework for both the document collection and validation of the findings derived from the SWOT analysis to ensure consistency with the established international frameworks. The SWOT analysis findings were refined for accuracy with support from the SMEs and were then used to inform policy recommendations. The SWOT findings were validated through a structured workshop involving SMEs who provided a consensus on the analysis. This validation process ensured that the final recommendations were evidence-based and aligned with stakeholder priorities and international standards. Ultimately, from this analysis, a tailored set of recommendations was developed to serve as a blueprint for refining Jordan’s policies, strategies, and practices. These recommendations are grounded in the evidence and best practices identified through benchmarking.

## 3. Results

### 3.1. One Health Approach to Zoonotic Diseases

Jordan acknowledges the importance of the One Health approach, which encompasses human, animal, and environmental health. However, challenges related to the One Health committee’s functioning have been identified, highlighting the need for more comprehensive guidelines, especially concerning zoonotic diseases such as monkeypox and brucella. These challenges underscore the importance of effective communication and collaboration among the different sectors involved in public health and disease prevention. This subsection summarizes the findings on the One Health Approach to zoonotic diseases in Jordan based on the desk reviews and KIIs. Table 1 summarizes the following documents:

- The Regulatory Framework: Jordan’s Public Health Law No. 47 (2008) includes provisions that align with the One Health principles, such as zoonotic disease control, environmental health, and infectious disease outbreak response. However, the law lacks explicit mention of the “One Health” concept. However, JCDC Bylaw No. 112 (2020) mandates the multisectoral coordination and integration of public health data. Various ministries govern aspects related to zoonotic diseases, food safety, and water quality, collectively aligning with the One Health principles;
- Governance and Coordination: The Ministry of Health (MOH) has established a dedicated One Health department and committee to monitor zoonotic diseases, develop treatment protocols, and disseminate information across sectors;
- Guidelines and Protocols: The existing guidelines, like the “Animal-Human Shared Diseases Guide” (2006) and the “Emerging and Reemerging Diseases Guide” (2009), are valuable resources but require updates to reflect new zoonotic diseases and the evolving best control practices.

### 3.2. Antimicrobial Stewardship (AMS)

This subsection summarizes the findings on AMS in Jordan based on desk reviews and KIIs. Table 1 summarizes the following documents:

- The Regulatory Framework: Jordan’s Public Health Laws, particularly Chapter Seven of Public Health Law No. 47 (2008), establishes a strong regulatory framework for pharmaceutical safety but lacks explicit regulations addressing Antimicrobial Resistance (AMR). The existing regulations focus primarily on drug safety and quality, with limited attention to responsible antimicrobial use and monitoring. Additionally, JCDC Bylaw No. 112 (2020) mandates overseeing the development and implementation of AMR control strategies, as well as coordinating AMR monitoring initiatives;
- Strategies and Plans: The National Action Plan for Combating AMR outlines objectives such as education, sanitation, and antibiotic use optimization. The plan is supplemented by an operational blueprint emphasizing law enforcement, treatment guideline development, public awareness campaigns, and sustainable funding. The MOH’s strategic plan (2023–2025) also addresses AMR through improved readiness and response mechanisms. The Jordan CDC strategy (2023–2025) addresses the prioritization of AMR as well;
- Guidelines and Policies: Several policies and protocols are in place, including the Policy on the Prophylactic Use of Antibiotics in Surgical Procedures (2022), the Auto-Stop Medication Orders Policy (2022), and Protocols for the Diagnoses, Treatment and Management of Hospital-Acquired Pneumonia (HAP) and Ventilator-Associated Pneumonia (VAP) (2023). However, monitoring and evaluation mechanisms are limited.

### 3.3. Climate Change

Climate change poses significant challenges to ecosystems, economies, public health, and social stability. This subsection summarizes the findings on climate change in Jordan based on desk reviews and KIIs. Table 1 summarizes the following documents:

- The Regulatory Framework: Jordan’s Environmental Protection Law (Law No. 6 of 2017) provides a robust framework for addressing climate change, pollution prevention, resource conservation, waste management, and environmental impact assessments;
- Strategies and Plans: The National Green Growth Strategy (2017) and the Ministry of Environment’s (MOE) Strategic Plan (2020–2022) integrate green development principles into government policies. The MOH’s strategic plan (2023–2025) focuses on adaptability, crisis management, and climate-related health impacts;
- Guidelines and Policies: Jordan’s guidelines for Environmental Impact Assessments (EIAs) for wastewater treatment plants (WWTPs) consider climate change impacts, evaluating resilience to climate events, and optimizing energy use. Policies to reduce plastic pollution are also in place but require further updates and research.

### 3.4. SWOT Analysis

The SWOT analysis below presents a structured overview of the strengths, weaknesses, opportunities, and threats related to the policies and legislations in relation to the One Health approach to zoonotic diseases, AMS, and climate change in Jordan. This analysis was derived from a situational analysis and benchmarking exercise, conducted after the desk review and KIIs. Please see Table 2.

## 4. Discussion

Our findings revealed that Jordan has made substantial steps in integrating the One Health approach to zoonoses, AMS, and climate change policies and strategies. These efforts align with both regional and international standards and highlight Jordan’s commitment to global public health. In the discussion, we provide a detailed elaboration on these efforts, discussing areas of strengths and opportunities for further enhancements.

### 4.1. One Health Approach to Zoonotic Diseases

Zoonoses account for more than 75% of EIDs in the modern era. Even though there have been notable recent advancements in clinical diagnostic techniques, medical practices, and environmental and epidemiological surveillance, zoonotic EIDs are still a serious global public health concern and are becoming more prevalent, particularly in less developed contexts. In 2016, the WHO advised Jordan to establish a national control strategy that gives priority to zoonoses [29].

Jordan’s One Health approach emphasizes the interconnection of human, animal, and environmental health, which is crucial for managing zoonotic diseases, and more research in Jordan has been conducted for approaching zoonoses using the One Health approach [2,3,30]. Additionally, regulatory frameworks such as Public Health Law No. 47 (2008) and JCDC Bylaw No. 112 (2020) provide a strong legal foundation for addressing zoonotic diseases. Jordan prioritizes diseases like brucellosis, salmonella, and MERS-CoV, which are significant in both regional and global contexts. While laws and regulations are in place to manage these diseases, such as the “Measures to Control the Spread of Infectious and Epidemiological Animal Diseases”, they often lack specific reporting mechanisms and MOH involvement. However, the desk review revealed that publications on One Health, such as the 2017 Joint External Evaluation of IHR Core Capacities, have pointed out several gaps in public health law. Moreover, there may be limitations in the legal framework’s ability to effectively address communicable diseases, which are currently only addressed in response to national-level events. By addressing these issues, Jordan could better align its legal framework with the One Health model and strengthen its preparedness for public health emergencies.

Jordan’s One Health approach is reinforced by guidelines like the “Animal-Human Shared Diseases Guide” and the “Emerging and Reemerging Diseases Guide”. These efforts align with the WHO’s One Health initiative, which emphasizes integrated disease management strategies [4]. Regionally, similar initiatives are seen in countries like Saudi Arabia and Egypt. For instance, Saudi Arabia’s Ministry of Health has employed extensive surveillance systems for zoonotic diseases, which Jordan could emulate to strengthen its own surveillance capabilities [31].

Internationally, countries like the United States of America (U.S.A.) have endorsed comprehensive One Health strategies that include detailed guidelines and multisectoral collaborations. These strategies highlight the importance of a national integrated surveillance system and the need for continuous updates to guidelines, areas where Jordan can develop its efforts [6]

The WHO’s guide “Taking a Multisectoral, One Health Approach: A Tripartite Guide to Addressing Zoonotic Diseases in Countries”, underscores the need for collaboration between the human, animal, and environmental health sectors to effectively manage zoonotic diseases. Jordan’s policies align with this approach by fostering intersectoral coordination through the JCDC and other governmental bodies [32]. Addressing the One Health approach to zoonotic diseases challenges requires an increase in infrastructure, capacity building, and policy development investments to support integrated surveillance systems [6].

### 4.2. Antimicrobial Stewardship (AMS)

The impact of AMR on a global scale is significant. The latest estimates from the Global Research on Antimicrobial Resistance (GRAM) project indicate that AMR was directly responsible for over 1.27 million deaths in 2019 and contributed to more than 4.95 million deaths worldwide, surpassing the combined mortality of HIV/AIDS and malaria [33]. Many countries and international organizations have acknowledged the crucial significance of AMS and have dedicated substantial resources to developing and implementing national and global strategies to combat AMR.

Like other countries, Jordan faces the challenge of AMR, as the WHO has reported a high occurrence of AMR in Jordan. To address this issue, the Jordanian government has taken measures to combat AMR and enhance AMS at the national level. In 2017, the Jordanian Ministry of Health (MOH) launched the National Action Plan for Combating Antimicrobial Resistance, which provides a comprehensive strategy to tackle this public health threat [34]. Notable initiatives include the implementation of AMS programs at healthcare facilities, such as hospitals and primary care clinics, to promote the appropriate use of antimicrobials.

The Jordan Food and Drug Administration (JFDA) is the principal national regulatory body in Jordan, tasked with safeguarding the safety, effectiveness, and quality of pharmaceuticals and food products. In Jordan, there are many AMS initiatives that are driven by the National Action Plan for Combating AMR and reinforced by legislative measures like Public Health Law No. 47 (2008) and the Pharmacy and Medicines Law of 2013. These efforts are critical given the global threat posed by AMR, which the WHO has identified as one of the most urgent public health threats [35].

Additionally, the government has implemented infection prevention and control measures, including hand hygiene, environmental cleaning, and the use of personal protective equipment, to minimize the spread of drug-resistant organisms [36]. To effectively monitor and combat AMR, Jordan has enhanced the capacity of its national surveillance systems. These technologies enable the monitoring of the AMR trends and resistance patterns and antimicrobial usage. Furthermore, educational and awareness programs have been implemented, targeting healthcare professionals, policymakers, and the public. These campaigns emphasize the importance of responsible antibiotic use and the risks associated with AMR [34,36]. Moreover, the government is actively promoting research and development initiatives to gain a better understanding of the local factors contributing to AMR. Additionally, there is a strong emphasis on fostering collaboration and coordination among various stakeholders, including the MOH, Ministry of Agriculture (MOA), MOE, academic institutions, and research organizations. This collaborative approach comprehensively addresses AMR, recognizing the interconnectedness of human, animal, and environmental health.

Moreover, Jordan has established the AMR Steering Committee and implemented a comprehensive AMR national action plan. These efforts are in line with the global standards and reflect Jordan’s commitment to tackling AMR. Regionally, countries like Egypt and Lebanon have also initiated national action plans focusing on strict regulations and public awareness campaigns to combat AMR [37,38]. The implementation of these regional AMR national action plans also faces challenges similar to those in Jordan’s implementation. These challenges include inadequate attention to responsible antimicrobial use and monitoring, insufficient public awareness, and inadequate regulations on veterinary antibiotic use. The data quality and utilization of AMR studies also need improvement.

Internationally, AMS programs in Europe and the United States (U.S.) are characterized by rigorous monitoring and evaluation mechanisms, which Jordan can adopt to improve its own systems. These programs emphasize the importance of comprehensive data collection, public awareness campaigns, and stringent regulatory measures to warrant the responsible use of antimicrobials [13].

The WHO’s “Antimicrobial Stewardship Programs in Health-care Facilities in Low-and Middle-income Countries: A WHO Practical Toolkit” provides practical guidance for implementing AMS programs. This toolkit focuses on core components, including improving antimicrobial use, enhancing infection prevention, and promoting education and training. Jordan’s AMS initiatives are aligned with these guidelines through its national action plan and public awareness campaigns.

### 4.3. Climate Change and Health

The UN has designated the Middle East and North Africa (MENA) region, where Jordan is located, as a hotspot for climate change, recognizing it as a key area. Scientific modeling consistently demonstrates that there is a concerning pattern of rapid warming in the region. The projected temperature rises are expected to be twice as high as the global average [22]. The climates vary greatly within the MENA region, and it is expected that the EMR will experience more increased warming trends than the global annual mean warming, with a predicted increase of 2.2 to 5.1 degrees Celsius [22].

At the same time, the MENA has the fewest renewable water resources and the least arable land per person of any region in the world. Climate models predict a significant reduction in precipitation in the majority of the Mediterranean, Northern Saharan, and EMR regions, ranging from 10-30% by the next century [21]. Although Jordan has made significant progress in declaring its official perspective on climate change, translating this into a unified and long-term policy framework remains obstructed.

Jordan’s climate change policies are integrated into broader environmental strategies, such as the National Green Growth Strategy and Environmental Protection Law No. 6 (2017). These frameworks address the health impacts of climate change, which is a critical issue for the region due to its extreme weather patterns and water scarcity. Jordan’s efforts include the National Climate Change Health Adaptation Strategy, which aligns with international sustainability objectives. This strategy emphasizes the need for a multisectoral approach to address the health impacts of climate change, including the integration of climate change considerations into health risk management strategies [15]

Regionally, countries like Morocco and Tunisia have developed national adaptation plans that include specific health strategies, which Jordan can benefit from to strengthen its own initiatives. These plans emphasize the importance of emergency preparedness and the integration of climate change considerations into public health policies [39,40].

Internationally, innovative climate health adaptation plans in countries like Germany and Canada emphasize comprehensive emergency preparedness and public health responses, which can serve as models for Jordan [41,42]. These countries highlight the need for dedicated climate change focal points in health ministries, improved data collection, and ongoing research to inform policy decisions [16].

The WHO’s “Compendium of WHO and Other UN Guidance on Health and Environment—Climate Change” provides comprehensive guidance on integrating health considerations into climate change policies. This compendium highlights the importance of multisectoral collaboration and evidence-based strategies. Jordan’s National Climate Change Health Adaptation Strategy aligns with these recommendations. However, monitoring protocols for greenhouse gas emissions and pollutants is insufficient, and there is a need for increased research and data collection to enlighten policy decisions.

### 4.4. Recommendations

To address the challenges identified in the national disease prevention mapping study in Jordan, it is crucial to implement a comprehensive and integrated approach across three key areas: One Health approach to zoonotic diseases, AMS, and climate change. The One Health framework should be comprehensively integrated into Jordan’s regulatory and operational structures to address zoonotic disease threats effectively. Legal and regulatory updates to Jordan’s public health laws are recommended to formalize the One Health approach, bridging the human, animal, and environmental health domains. A reinforced multisectoral coordination mechanism should also be established, enhancing collaboration between the MOH, MOA, and other stakeholders to streamline disease surveillance and response efforts.

Updated guidelines for zoonotic disease management are critical and should reflect contemporary global standards for disease prevention and control. Additionally, public awareness campaigns should be launched to educate communities on zoonotic risks and preventive practices, fostering a culture of health vigilance and preparedness. To support the sustained implementation of One Health initiatives, stable funding sources must be secured, ensuring resilience in Jordan’s response to zoonotic diseases.

In response to the growing threat of AMR, Jordan should prioritize a robust AMS program focused on regulatory reinforcement, education, and research. Pharmaceutical regulations should be strengthened to govern responsible antimicrobial use across human and animal sectors, with an emphasis on monitoring antibiotic distribution and application. To build awareness around AMR, targeted public education programs should be implemented, reaching healthcare providers, agricultural stakeholders, and the general public. Strengthening veterinary regulations is imperative to mitigate AMR in animal populations, an important reservoir for resistance transmission to humans. Additionally, improving the data infrastructure for AMR surveillance by standardizing data collection and facilitating real-time sharing will enhance Jordan’s capacity to monitor resistance trends and respond proactively. Investment in AMR-focused research is also essential, supporting the development of alternative treatments and diagnostic tools that address the local AMR dynamics. Finally, fostering multisectoral partnerships will enable collaborative AMS efforts, uniting resources and expertise to address AMR from multiple angles.

Addressing the health impacts of climate change requires that Jordan’s health policies incorporate climate resilience and emergency preparedness plans. This involves establishing protocols for managing health risks associated with climate variability, such as extreme weather events and vector-borne diseases, in the MOH’s risk management strategies. Enhanced monitoring and research protocols for greenhouse gas emissions and other pollutants are essential to inform evidence-based policies and adaptation strategies.

Investments in training health personnel for climate-related health risks will equip Jordan’s workforce with the skills necessary to address emerging climate challenges. Public sustainability education campaigns can further engage communities in climate resilience practices, supporting a health-conscious, environmentally aware society. To achieve Jordan’s climate objectives, increased support for renewable energy and conservation efforts is recommended, aligned with Jordan’s National Climate Change Health Adaptation Strategy. Expanding international partnerships will bring in the necessary resources, expertise, and funding, facilitating knowledge exchange and enabling Jordan to benefit from global advancements in climate adaptation.

## 5. Conclusions

Jordan’s integration of the One Health approach to zoonotic diseases, AMS, and climate change adaptation into its disease prevention policies is commendable and aligns with global health priorities. However, while Jordan’s efforts are commendable, there are areas that could be further strengthened to enhance the effectiveness of these initiatives. Updating the public health law and relevant guidelines and policies is a crucial step in ensuring that the legal and regulatory frameworks are up-to-date and fully supportive of modern, integrated approaches like One Health. This includes revising laws that govern animal health, environmental protections, and antimicrobial usage to ensure they are comprehensive. Strengthening and structuring public awareness campaigns is equally important, as these campaigns help to ensure that the general population, healthcare providers, and policymakers understand the interconnections between human, animal, and environmental health and are equipped to respond effectively to emerging health threats. Public engagement and education can also play a critical role in promoting behavioral changes that reduce the spread of zoonotic diseases or encourage the responsible use of antimicrobials.

In addition, Jordan can enhance its efforts by developing detailed climate change adaptation strategies that integrate health considerations. As climate change increasingly influences disease dynamics, Jordan must plan for these evolving risks. This can be accomplished by strengthening climate-sensitive disease surveillance systems, improving the resilience of public health infrastructure, and creating policies that support climate adaptation in both urban and rural areas. Investment in infrastructure, particularly in healthcare systems, veterinary services, and environmental monitoring, will be critical for sustaining long-term disease prevention strategies. Moreover, capacity building within Jordan’s public health, veterinary, and environmental sectors will ensure that key stakeholders have the skills and resources necessary to implement and manage integrated One Health policies effectively. Moreover, fostering collaboration between the public health, animal health, and environmental sectors, as well as investing in education, research, and technology to stay ahead of emerging health threats, are pivotal. By strengthening the policies and investment in these areas, Jordan will be better equipped to meet the health challenges and contribute to global efforts in controlling zoonotic diseases, managing antimicrobial resistance, and mitigating the impacts of climate change on public health.

## Figures and Tables

**Table 1:** Key documents related to One Health approach to zoonoses, antimicrobial stewardship, and climate change in Jordan *.

**Component**	**One Health Approach to** **Zoonotic Diseases**	**Antimicrobial Stewardship**	**Climate Change**
Regulatory Framework	- Comprehensive laws like Public Health Law No. 47 (2008)—article five	- Public Health Law No. 47 (2008)—article seven - Pharmacy and Medicines Law of 2013 - Specific policies like Auto-Stop Medication Orders Policy (2022)	- Environmental Protection Law No. 6 (2017)
Governance and Coordination	- MOH and MOA dedicated departments - Interdisciplinary One Health committee - Establishment of Jordan Center for Disease Control mandated with multisectoral coordination.	- AMR Steering Committee - Dedicated Pharmacy Directorate - Jordan Center for Disease Control Bylaw No. 112 (2020) mandates overseeing the development and implementation of AMR control strategies, coordinating AMR monitoring initiatives.	- Ministry of Environment coordination - Lack of climate change focal point in MOH
Strategies and Plans	- The concept is integrated within broader healthcare strategies - No explicit One Health approach to zoonotic disease strategies	- National Action Plan for Combating AMR (2018–2022) - MOH strategic plan (2023–2025)	- National Green Growth Strategy (2017) - MOE Strategic Plan (2020-2022) - Climate Investment and Mobilization Plan (2022) - National Climate Change Health Adaptation Strategy and Action Plan (2012)
Guidelines and Policies	- Animal-Human Shared Diseases Guide (2006) - Emerging and Reemerging Diseases Guide (2009)	- Policy for Confronting Multi-Drug Resistance Bacteria (MDRO) - Policy on the Prophylactic Use of Antibiotics in Surgical Procedures (2022) - Auto-Stop Medication Orders Policy (2022) - Protocols for Diagnoses, Treatment and Management if Hospital-Acquired Pneumonia (HAP) and Ventilator-Associated Pneumonia (VAP) (2023)	- Guidelines for Environmental Impact Assessments (EIA) - Policies on plastic pollution

* MOH: Ministry of Health; MOE: Ministry of Environment; MOA: Ministry of Agriculture.

**Table 2:** SWOT analysis.

$S_{\mathrm{trength}}$ **One Health Approach to Zoonotic Diseases** Presence of a legal framework Memorandum of Understandings for data sharing between MOH and MOA Dedicated departments and committees Prioritization of key zoonotic diseases International collaborations (quadripartite) **Antimicrobial Stewardship** Robust regulatory framework Detailed national action plans Active regulatory bodies **Climate Change** Comprehensive legal framework Alignment with national and international sustainability objectives Emphasis on environmental preservation	$W_{\mathrm{eaknesses}}$ **One Health Approach to Zoonotic Diseases** Lack of explicit One Health definitions Insufficient collaboration Need for updated guidelines **Antimicrobial Stewardship** Limited focus on antimicrobial use and monitoring Inadequate public awareness Limited veterinary regulations Limited monitoring and evaluation mechanisms Insufficient engagement of MOE and MOWI **Climate Change** Absence of emergency and preparedness plan Lack of assigned/dedicated climate change focal points in different ministries Inadequate monitoring protocols Insufficient multisectoral collaboration
$O_{\mathrm{pportunities}}$ **One Health Approach to Zoonotic Diseases** Enhance reporting mechanisms Strengthen collaboration Develop targeted strategies **Antimicrobial Stewardship** Revise regulations Implement public awareness campaigns Secure sustainable funding **Climate Change** Establish emergency preparedness plans Integrate climate change into health risk strategies Expand international collaborations	$T_{\mathrm{hreats}}$ **One Health Approach to Zoonotic Diseases** Potential gaps in enforcement Resource constraints Bureaucratic delays **Antimicrobial Stewardship** Easy availability of antibiotics Lack of unified multisectoral data Inadequate financial allocation **Climate Change** Insufficient integration of climate change considerations Insufficient political commitment Emerging evidence needs incorporation

## Data Availability

All data supporting the findings of this study are available within the paper and its Supplementary Information.

## Supplementary Materials

The following supporting information can be downloaded at https://www.mdpi.com/article/10.3390/ijerph22050749/s1, File S1: List of key stakeholders; File S2: Interview guide.
