# Supplementary material for: Exploring Policies, Strategies, and Legislations Related to the One Health Approach to Zoonoses, Antimicrobial Stewardship, and Climate Change in Jordan: A Multimethod Study with SWOT Analysis
Source: Int J Environ Res Public Health. 2025 May 9;22(5):749. doi: 10.3390/ijerph22050749 (PMC12111497; doi:10.3390/ijerph22050749)
Supplement: Supplementary file 1 [file ijerph-22-00749-s001.zip › ijerph-3270000-supplementary/S1- Key Stakeholders.docx]

**SUPPLEMENTARY FILE 1: LIST OF KEY STAKEHOLDERS**

| **Entity** | **Stakeholder Roles** |
| --- | --- |
| Ministry of Health (MoH) | - Director of Communicable Disease Directorate |
|  | - Head of Epidemiology Department |
|  | - Senior officials from Infection Control and Prevention |
|  | - Director of Environmental Health Directorate |
|  | - Head of Vaccination and Serum Department |
| Royal Medical Services (RMS) | - Public health and infection control leaders |
| Ministry of Agriculture (MoA) | - Director of Animal Wealth Directorate |
|  | - Veterinary and zoonotic disease experts |
| Ministry of Environment (MoEnv) | - Director of Environmental Inspection and Monitoring |
| Jordan Food and Drug Administration (JFDA) | - Director of Drug division  - Director of Food division |
| Ministry of Water and Irrigation (MOWI) | - Deputy Secretary General |
| Amman Municipality | - Director of Health and Occupational Safety |
| Ministry of Labor | - Director of Occupational Safety and Health |
| National Center for Security and Crisis Management | - Public health and emergency planning officials |
| International Organizations | - WHO Representative |
|  | - UNICEF Representative |
|  | - FAO Representative |
|  | - UNHCR Representative |
|  | - IOM Representative |
| Non-Governmental Organizations (NGOs) | - Healthcare and public health advocacy representatives |
| King Hussein Cancer Center (KHCC) | - Head of Infection Control |
| Jordan University of Science and Technology (JUST) | - Public health consultant |
| UNRWA | - Health services representative |
