# Supplementary material for: Exploring Policies, Strategies, and Legislations Related to the One Health Approach to Zoonoses, Antimicrobial Stewardship, and Climate Change in Jordan: A Multimethod Study with SWOT Analysis
Source: Int J Environ Res Public Health. 2025 May 9;22(5):749. doi: 10.3390/ijerph22050749 (PMC12111497; doi:10.3390/ijerph22050749)
Supplement: Supplementary file 1 [file ijerph-22-00749-s001.zip › ijerph-3270000-supplementary/S2- Interview Guide.docx]

**SUPPLEMENTARY FILE 2: INTERVIEW GUIDE TOOL**

1. **Participants background information:**
2. Name of the organization/institution the participant represents
3. Participant's role/position within the organization
4. Years of experience in disease prevention and control
5. **Existing Policies & strategies regarding “disease prevention related to epidemics, communicable and zoonotic diseases within the One Health, AMR, vaccination, respiratory diseases, WASH, RCCE, IPC, HAI, and health-related environmental threat’’. *** Depending on the area of the stakeholder interviewed**
6. Please may you provide us with any existing Policies & strategies available?
7. What do you think the notable gaps or challenges in the current policies, legislation and strategies?
8. May you describe in detail any recent updates or revisions to these policies, legislation and strategies?
9. **Existing legislations, guidelines, protocols, SOPs & assessments regarding “disease prevention related to epidemics, communicable and zoonotic diseases within the One Health, AMR, vaccination, respiratory diseases, WASH, RCCE, IPC, HAI, and health-related environmental threats”. *** Depending on the area of the stakeholder interviewed**
10. Please provide us with any specific legislation related to the above.
11. Are there guidelines or standard operating procedures (SOPs) that accompany these legislations? If yes, please describe them
12. How are these legislations, guidelines and protocols enforced and monitored for compliance?
13. What are the challenges or barriers in implementing and enforcing these legislations, guidelines and SOPs?
14. **Implementation and Monitoring**
15. What mechanisms are in place to ensure the implementation of disease prevention measures outlined in the policies, strategies, legislations, guidelines, and SOPs?
16. How are responsibilities assigned and coordinated among different stakeholders for the implementation of these measures?
17. Are there any monitoring and evaluation procedures to assess the effectiveness of these disease prevention measures?
18. How frequently are these measures reviewed and updated based on monitoring and evaluation results?
19. **Intersectoral Collaboration**
20. Are there collaborations or partnerships between different sectors (e.g., health, environment, agriculture) to address One Health-related challenges? Please explain how these collaborations are managed
21. How do these collaborations contribute to disease prevention efforts?
22. Please provide us with any challenges or successes in fostering collaboration across sectors.
23. **May you provide and count any Challenges, Lessons Learned and Best Practices**
24. **Any recommendations / additional comments**
